# Non-apoptotic Caspase regulation of stem cell properties

**DOI:** 10.1016/j.semcdb.2017.10.034

**Published:** 2018-10

**Authors:** Luis Alberto Baena-Lopez, Lewis Arthurton, Derek Cui Xu, Alessia Galasso

**Affiliations:** University of Oxford, Sir William Dunn School of Pathology, Oxford, OX13RE, United Kingdom

**Keywords:** Apoptosis, Caspases, Caspase treatment, Non-apoptotic functions, Stem cell

## Abstract

The evolutionarily conserved family of proteins called caspases are the main factors mediating the orchestrated programme of cell suicide known as apoptosis. Since this protein family was associated with this essential biological function, the majority of scientific efforts were focused towards understanding their molecular activation and function during cell death. However, an emerging body of evidence has highlighted a repertoire of non-lethal roles within a large variety of cell types, including stem cells. Here we intend to provide a comprehensive overview of the key role of caspases as regulators of stem cell properties. Finally, we briefly discuss the possible pathological consequences of caspase malfunction in stem cells, and the therapeutic potential of caspase regulation applied to this context.

## Highlights

• General overview of the caspase protein family.
• Stem cells: fundamental concepts.
• Caspase roles in embryonic and Induced pluripotent stem cells.
• Caspase roles in adult stem cells.
• Pathological consequences of caspase deregulation and therapeutic potential of caspase modulation.

## 1 General overview of the caspase protein family

**Fig. 1:**
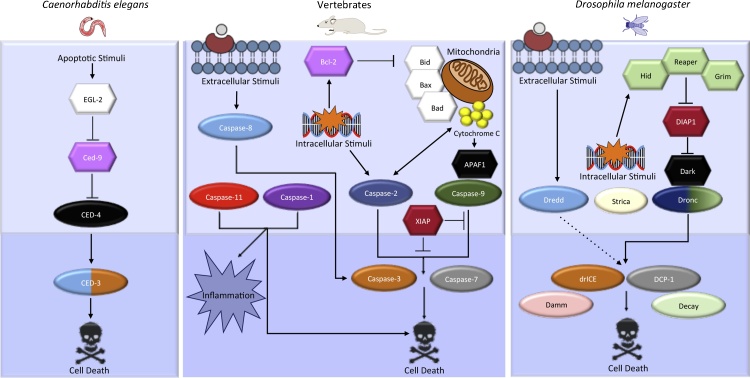
Diagram showing the evolutionary conservation of the main caspase regulators of apoptosis. The ellipsoid shape designates all caspase members included in the apoptotic pathway, whereas the hexagons are accessory apoptotic proteins. Similar proteins across species follow the same colour scheme. The light blue region encompasses what are considered the apical/initiator caspase members, whereas the dark blue area sorrounds the effector/executioner caspases.

In nature, being in the wrong place often has fatal consequences. At the cellular level, this situation normally ends with the elimination of misplaced elements via genetically encoded systems of programmed cell death. Caspases are Cysteine-ASPartic proteASES present in all metazoans that have been intensively studied for being the major regulators of programmed cell death through apoptosis [1], [2], [3], [4], [5], [6], [7], [8] (Fig. 1). They execute this biological function by utilizing their protease activity over a plethora of target substrates located in different subcellular organelles [7], which ultimately provokes a generalized collapse of all metabolic functions. Structurally, all caspases contain one large and one small subunit that form the catalytic pocket responsible for enzymatic function. Additionally, some members incorporate a large pro-domain of variable composition appended to the N-terminal end. Depending on the structural composition of the pro-domain – some of which include CAspase Recruiting or Death Effector Domains (CARDs and DEDs) – different adaptor complexes can interact with the several members of the caspase family [7], [8]. Historically, the caspase members have been grossly classified as apoptotic or inflammatory, taking into account their primary roles, but this classification does not accurately reflect their diverse functional nature, since several caspases can participate in both of these processes, as well as others [8]. At least in the context of apoptosis, it is more precise to classify the caspases as initiator/apical and executioners/effector caspases, based on their early or late activation during this process [8] (Fig. 1).

Synthesized as inactive zymogens, caspases only become fully active after several steps of self-processing upon multimerization [8], [9], [10]. Initiator caspases are responsible for the exponential activation of effector caspases during apoptosis [11]. Complex signalling events arising from intracellular organelles (mainly mitochondria) and/or extracellular receptors facilitate the engagement of multimeric adaptor platforms (apoptosome, inflammasome, piddosome) that promote caspase activation [8]. Conversely, caspases are inactivated through either post-translational modifications − mainly phosphorylation and ubiquitylation − or interactions with modulatory proteins [6], [9], [10], [12], [13]. Stringent regulation of caspase activation is crucial to avoid the inadvertent activation of cell death as well as an onset of diseases [1], [2], [8], [14]. Beyond this well-characterized apoptotic role, recent investigations have shown that moderate levels of caspase activation can transiently process localized substrates in specific subcellular compartments without causing cell death [2], [3], [4], [5], [6], [15]. For example, it has been reported that moderate caspase activity in neuronal dendrites is crucial to remodel such cellular projections without causing apoptosis, thus reconfiguring the network of neural connectivity [16]. These non-lethal activities are able to modify the function of binding partners and substrates, often stimulating their degradation, but also triggering their subcellular delocalization, activation or differential binding to other proteins. Indeed, the non-lethal caspase activities can be exclusively mediated by protein–protein interactions without the need for enzymatic function (e.g. [17]). Importantly, non-lethal caspase activation has been shown to be instrumental in controlling a broad range of essential cellular processes (e.g proliferation, cell fate determination, differentiation, migration, secretion, cytoskeleton remodelling [1], [2], [3], [4], [5], [6], [8]) in a tissue-specific manner. Furthermore, if deregulated, they can contribute decisively to the pathophysiology of multiple diseases [1], [3], [5], [8], [18], [19], [20], [21], [22], [23], [24]. Although our current knowledge concerning the biochemistry of caspase activation is quite detailed during apoptosis, much remains unknown in non-lethal contexts. Furthermore, the identity of tissue specific target substrates participating in these non-apoptotic functions, remains elusive. The elucidation of these questions is essential to fully understand caspase biology, and potentially develop efficient therapeutic interventions against caspase-associated diseases.

## 2 Stem cells: fundamental concepts

**Fig. 2:**
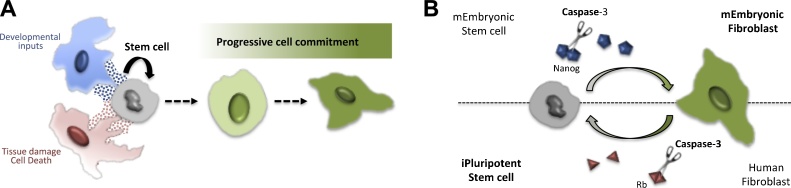
Schematic representation of stem cell functions and examples of caspase regulation in Embryonic Stem Cells (ESCs). A) Basic representation of stem cells properties. Naïve stem cells are represented in grey. Stem cells can self-renew or differentiate into specific cell derivatives of the resident tissue. Cells normally in close proximity to the stem cells (blue and red cells) are able to provide essential signals (represented by blue and red dots) to the stem cells via soluble factors or cellular contacts. B) Reciprocal examples of caspase regulatory roles in pluripotent stem cells. Caspase activity (scissors) facilitates the enzymatic processing of key transcription factors essential to promote the differentiation of ESCs (Nanog), as well as reprograming differentiated cells into induced pluripotent cells (Rb).

One of the most remarkable achievements in the biology of multicellular organisms is the sophisticated variety of differentiated cells and tissues generated from a single primordial cell throughout development. Equally astonishing is the enduring potential for regeneration present in most organs, which protects against the cellular wear and tear triggered by various intrinsic or environmental insults. Both scenarios demand the presence of undifferentiated cellular precursors with the capacity for self-renewal and differentiation, known as stem cells [25], [26] (Fig. 2A). In this review, we intend to provide a comprehensive compilation of the most recent findings that associate the non-lethal activity of caspases with the regulation of stem cell physiology.

The self-renewing capability and the competency to acquire multiple cell fates are the basic properties defining a stem cell. Several types of stem cells, distinguishable by their cellular ontogeny and differentiation potential, have been described. Embryonic stem cells (ESCs) are present in early embryos (usually before pre-implantation) and possess unlimited differentiation potential (totipotent or pluripotent). They can therefore give rise to virtually any cell type of the organism [25]. Adult or somatic stem cells retain the ability to self-renew, but are restricted in their differentiation potential to those cell derivatives existing within its host tissue. Adult stem cells can show either unipotent or multipotent differentiation capabilities, if they can give rise to one or several cell types of the resident tissue, respectively [26]. Importantly, somatic stem cells are responsible for maintaining and repairing their host tissues upon demand, remaining most of the time in quiescence [26] (Fig. 2A). The exit from quiescence (reactivation of proliferation and differentiation) relies heavily on complex cellular interactions established between the stem cells and the surrounding differentiated cells, which ultimately form the so-called stem-cell niche [26], [27] (Fig. 2A). In each particular tissue, intricate signalling networks allow the efficient intercellular communication between stem cells and the surrounding cellular microenvironment [27]. This communication relies on soluble factors [28], [29] and on cell contact interactions [27] (Fig. 2A).

In recent years, the development of protocols to reprogram adult stem cells into pluripotent stem cells (induced pluripotent cells, iPSCs) has brought about the opportunity to obtain large numbers of stem cells in culture conditions [26]. These protocols mimic the transcriptional network (Nanog, Oct4, Sox2, c-Myc and Kif4) [30] that promotes self-renewal and represses differentiation, thereby maintaining pluripotency in ESCs. These stem cell-promoting factors can also reset the genetic programs acquired during development in adult cells, whilst conferring pluripotency [31]. The usage of iPSCs clinically for regenerative purposes has not been fully accomplished but already represents one of the major breakthroughs for obtaining personalized medicine. Interestingly, some of the aforementioned transcriptional regulators contain caspase-cleavage sites that, upon proteolytic processing, permanently modify their activities [32]. The latter is probably one of the most powerful arguments to include the caspases within the list of key factors involved in the regulation of stem cell properties.

Unfortunately, deregulation of stem cell properties is often the underlying cause of human diseases, such as neurodegeneration, immune disorders and cancer. Recent investigations have coined the concept of cancer stem cells to refer to those proliferative cells within tumours with higher resistance to chemotherapy [33]. It is accepted that these cells facilitate tumour relapse and propagation, thus becoming a major obstacle for cancer treatment. A better understanding of the mechanisms that regulate stem cell functions − and by extension the potential links with caspase activity – could help to identify the origins of different diseases, as well as new therapeutic interventions.

## 3 Caspase roles in embryonic and induced pluripotent stem cells

One of the seminal observations suggesting a potential regulatory role for caspases in embryonic stem cell physiology was obtained in 2002, through the phenotypic analysis of caspase-8 mutant mice. The work of K. Sakamaki and collaborators showed that animals deficient in caspase-8 die at early stages of development with severe morphological defects in the neural tube. Remarkably these phenotypes were not correlated with apoptotic flaws, but instead were attributed to a reduced expression of neurogenic markers and aberrant differentiation of neural precursors [34]. However, the functional confirmation of the relation between caspases and ESCs would not appear until 2008 [32]. At that time, the work of Fujita and collaborators showed the inability of murine-embryonic stem cells (mESCs) mutant for caspase-3 and caspase-9 to differentiate [32] (Fig. 2B). Further confirmation of these observations was obtained using specific caspase inhibitors able to block the enzymatic activity of these proteins. In addition it was shown that specific mutations in the stem cell factor Nanog were able to impede its caspase-dependent degradation and subsequently, the process of cell differentiation [32] (Fig. 2B). Importantly the caspase cleavage sites required for Nanog degradation in mice are conserved in the human homologue of this protein [32].

Human-embryonic stem cells (hESCs) and iPSCs in culture are subject to constant anoikis and cell differentiation in the absence of basic fibroblast growth factor (bFGF) [35]; anoikis is an specialized version of programmed cell death observed in anchorage-dependent cells that occurs following detachment from the extracellular matrix. Caspase-3 inhibition can largely prevent the induced-apoptosis as well as the excess of cell differentiation in this cellular scenario, but the cellular detachment still persists [35]. Interestingly, the cleavage of Nanog mediated by Caspase-3 is one of the key factors facilitating the differentiation observed in hESCs and iPSCs deprived of bFGF [35]. Additionally it has been reported that chemical compounds with Rock-inhibitory activity generate similar phenotypes, by blocking the caspase-ROCK1-myosin signalling pathway [36].

Recent studies suggest that sublethal caspase activation in murine-ESCs promotes the proliferation and differentiation of c-Kit^+^/α-actinin^low^ cardiac progenitor cells [37]. Reciprocally, caspase inhibition dramatically reduces the amount of differentiated cardiomyocytes [37]. In addition, silencing of caspase-3 and caspase-7 in murine models depletes the numbers of differentiated myocytes in the developing heart [38]. Interestingly, it has been suggested that the level of cytochrome *c* released from the mitochondria overtime could define the level of caspase-3 activation, which is instrumental for discriminating between the apoptotic effects and the differentiation functions of mESCs [39].

In contrast to caspase functions in ESCs, the activation of caspase −3 and −8 is also key to revert the differentiation of human fibroblast for obtaining iPSCs [40] (Fig. 2B). This process requires the caspase-mediated degradation of the Retinoblastoma protein (Rb). Furthermore, uncleavable mutant versions of the Rb factor inhibit iPSC induction more efficiently than the wild type form of the protein [40] (Fig. 2B). Not surprisingly, the peak in expression of caspase-3 and −8 that precedes the reprogramming process, largely depends on the presence of the stem cell factor Oct-4 [40]. The dual role of caspases as drivers of de-differentiation in adult stems cells and differentiation in ESCs suggests the intriguing possibility that they can act as bimodal regulators. Depending on the repertoire of substrates or binding partners available in each cellular context, these enzymes could control the directionality of the cell differentiation programme and in general terms cell plasticity. Future investigations should illuminate the molecular basis of this duality, as well as the nature of the upstream signals triggering caspase activation in each cellular scenario.

Altogether the experimental evidence suggests the inhibition of caspase activation in pluripotent stem cells could have broader implications than preventing apoptosis. Indeed the usage of caspase inhibitors may be a powerful tool to fine-tune the differentiation status and reprogramming potential of ESCs and iPSCs, respectively. Unsurprisingly, the caspase-mediated regulation of pluripotency is evolutionarily conserved [41]. The work of Weaver and colleagues implicated the caspase member of *Caenorhabditis elegans,* CED-3, in the processing of key pluripotency factors (LIN-14, LIN-28 and DISL2) that regulate a large cohort of miRNAs, and ultimately stem cell properties [41], [42].

## 4 Caspase roles in adult stem cells

**Fig. 3:**
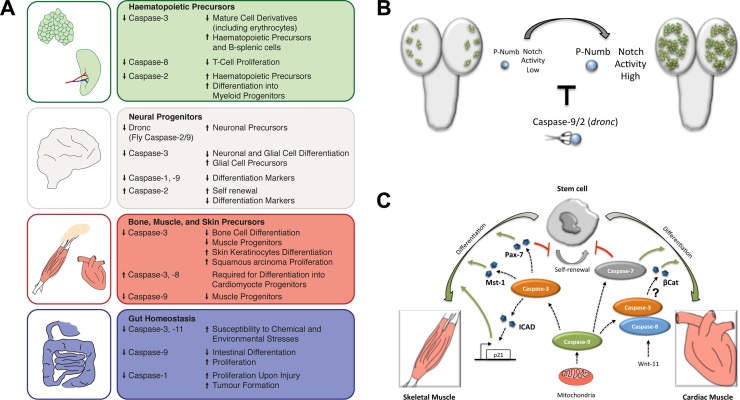
Caspase roles in adult stem cells. A) General overview of caspase functions in several tissues. Arrowheads preceding a particular caspase member indicate its level of actovation. Arrowheads preceding effects indicate either an increase or decrease of the subsequent description. B) The regulation of stem cell proliferation (neuroblast, green shapes) in the *Drosophila* brain (in grey) is mediated by protein-protein interactions involving caspases (scissor). Notch activity is regulated in *Drosophila* brain by the levels of phosphorylated Numb (p-Numb, blue round shape). High levels of p-Numb promote excess of cell proliferation in specific types of neuroblasts (green shapes). Protein-protein interactions of Droncwith p-Numb prevent uncontrolled neuroblast proliferation. C) Summary of specific caspase functions in muscles.

The majority of adult tissues contain a pool of undifferentiated precursors with the unlimited ability for self-renewal. Upon demand, adult stem cells can differentiate into one or several cell types of the host tissue. An imbalance between the rate of proliferation and differentiation of stem cells can instigate multiple diseases, and therefore sophisticated signalling mechanisms tightly control stem cell physiology. Most of our current knowledge linking the activity of caspases with the regulation of stem cell properties comes from the functional data obtained from different types of adult stem cells. In some cases the sublethal caspase activity drives the differentiation process of adult stem cells, while in others, it contributes to stem cell maintenance (Fig. 3A). The molecular mechanisms involved in these functions are highly tissue specific and therefore difficult to generalize. Since the primary aim of this review is to highlight the role of caspases in adult stem cells, descriptions of functions related with terminal cell differentiation have been largely omitted [43], [44], [45], [46].

### 4.1 Caspase roles in haematopoietic precursors

There is a body of evidence suggesting that caspases play a key role in regulating the properties of primordial hematopoietic precursors as well as lymphoid and myeloid derivatives (Fig. 3A). Adult hematopoietic stem cells have a limited sensitivity to cytokines and environmental stimuli that ensure their durability in quiescence. [47]. In contrast to normal conditions, the haematopoietic precursors in caspase-3 mutant mice exhibit premature exit from quiescence and overproliferation phenotypes without showing apoptotic defects [47]. Furthermore, silencing of caspase-3 is also correlated with inappropriate cell differentiation and deficit of mature cell derivatives within peripheral tissues [47] (Fig. 3A). At the molecular level, all these phenotypes were linked to the inability of caspase-3 to regulate the Ras-MAPK signalling pathway in response to specific cytokines, through molecular mechanisms not yet fully understood [47]. Interestingly recent data indicates that the combined reduction of the cell cycle inhibitor p21 and caspase-3 enhances the hyperproliferation phenotypes in the bone marrow, but also restores the presence of differentiated cell derivatives in peripheral tissues [48]. The authors speculate that such recovery in peripheral tissues could be related with sensitive changes to environmental cytokines in a double mutant condition for p21 and caspase-3 [48].

Caspase-2 deficient mice provide another example of the sublethal caspase roles in primary hematopoietic precursors. Caspase-2 deficiency results in a significant increase in the number of haematopoietic precursors, and skewed differentiation towards myeloid progenitors in aged mutant mice [49] (Fig. 3A). Phenotypically, these effects lead to increased numbers of granulocyte macrophages and erythroid progenitors over time. Intriguingly these phenotypes are also accompanied by frequent cell aneuploidy and tumour development, which could be indirectly facilitating differentiation defects [49].

Caspase-dependent phenotypes are not only observed in haematopoietic stem cells, but also frequently appear in more mature precursors resident in peripheral tissues. Splenic B-cells from caspase-3 deficient mice hyperproliferate through stable p21-PCNA protein complexes that recruit activated cell cycle regulators (CDK-cyclin complexes) [50] (Fig. 3A). These results were unexpected considering the antiproliferative role of p21 in most cell types [51]. Similarly the activity of caspase-8 is essential for T-cell proliferation upon cytokine stimulation [52], [53], perhaps through the proteolytic processing of p27 [54]. However the results obtained from in vivo and in vitro experiments are not equivalent on this regard [53], [54]. In contrast to caspase-3, caspase-8 deficient mice have a diminished rate of cell proliferation and colony formation of hematopoietic myeloid progenitors in response to specific mitogenic stimulus [55]. In addition it has been described that differentiation of monocytes into macrophages and dendritic cells also demands the activation of caspase-8 and caspase-3, respectively [56], [57]. Several molecular targets have been suggested to explain such caspase-dependent differentiation effects in myeloid derivatives (PARP, GATA-1 and acinus), however unambiguous experimental data supporting their implication is still lacking. The differentiation of red blood cells (erythrocytes) is one of the cellular scenarios better characterized at molecular level. In this context caspase-3 cleaves GATA-1, thus facilitating the erythrocyte differentiation [58], [59] (Fig. 3A). Remarkably, in contrast to the initial suggested caspase-differentiating role, current evidence indicates that caspase-3 activation is required to sustain the proliferation rate and timely differentiation of erythroid precursors [60]. Paradoxically, reduced caspase-3 activity in erythroblasts increases the susceptibility to cell death, likely through autophagy [60]. Altogether, the evidence emphasizes the divergent roles of different members within the caspase family, as well as the likely dependency of these functions on their molecular interactions with specific signalling pathways and target effector proteins.

### 4.2 The connection between caspases and neural progenitors

Model organisms such as *Drosophila melanogaster* and *Caenorhabditis elegans* offer powerful genetic tractability for uncovering the molecular details of fundamental biological processes, including stem cell regulation. The sensory organ precursors (SOPs) in flies have been meticulously studied over the years to understand many aspects of developmental biology and have provided detailed mechanistic insights regarding the instrumental role of caspases during neural stem cell differentiation. In this cellular model, it has been demonstrated that the proteolytic activity of the *Drosophila* homolog of caspase-2/9, *dronc*, is key for regulating the function of Shaggy/GSK3-β, and ultimately for preventing the specification of supernumerary sensory organ precursors [61]. After this initial discovery, the molecular regulation of *dronc* via the *Drosophila* inhibitor of apoptosis, DIAP1, and a *Drosophila* IKK-related kinase was described [62]. Furthermore, a recent report has shown that the *Drosophila* orthologue of the mammalian non-muscle myosin MYO7A (*crinkled*) acts as an adapter protein that brings together Dronc and the inactive form of Shaggy/GSK3-β, thus facilitating its activation through proteolytic processing [63]. Interestingly the functional role of Dronc in the regulation of neural precursors is not restricted to the peripheral nervous system, as it is also observed within type II neuroblast stem cells of the fly central nervous system [17]. Intriguingly, the catalytic activity of Dronc is not required in this cellular scenario; instead its effect relies on protein–protein interactions with the Notch signalling regulator, Numb [17] (Fig. 3B). More specifically, this interaction appears to be critical in limiting the activity of the phosphorylated version of Numb, which during overexpression leads to Notch signalling deregulation and unrestrained proliferation [17]. Beyond *Drosophila*, mammalian systems have also depicted the role of caspases as regulators of neural stem cell precursors. Indeed caspase-3 activity is crucial to modulate the differentiation of neural precursors in mice, through mechanisms that could involve the regulation of the cytoskeleton via PAK1 [64]. A similar requirement of caspase-3 activity has been described for timing the differentiation of neurons in the cerebellar cortex and Bergmann glial cells, respectively [65], [66] (Fig. 3A). In the latter, the lack of caspase-3 activity increases the number of glial cell precursors, while preventing its differentiation [66].

PC12 cells derived from rat pheochromocytoma are a widely used cellular system to study neural differentiation in culture [67]. The depletion of caspase-1 in these cells prevents the expression of differentiation markers such as Fodrin and Calpains [67]. These caspase targets are crucial in orchestrating the timely transition to differentiation of PC12 cells [67]. Similarly, human NT-2 cells represent a well-characterized cellular system used to study neural differentiation of cell progenitors upon treatment with retinoic acid [68]. The depletion of caspase-9 in these cells also prevents the expression of differentiation markers, likely due to defective processing of the class III histone deacetylase Sirt1 [68]. Interestingly, caspase-2 in the same cellular scenario plays an opposing role and impedes cell commitment through molecular mechanisms which remain unclear [68] (Fig. 3A). In addition to the reviewed information, caspases are instrumental for other biological processes (e.g. regulation of dendritic pruning, cell migration, etc…) within the nervous system, which have not been discussed as they are outside of the scope of this review [4], [15], [69].

### 4.3 Caspases as regulators of intestinal precursors homeostasis

The relation between caspases and intestinal homeostasis is highly complex, and is strongly linked to the regulation of inflammatory and apoptotic processes [70]. However there are also direct caspase-mediated non-lethal effects described in intestinal precursors. The high degree of cellular and functional conservation between the mammalian intestine and the *Drosophila* gut has incentivised the usage of this model system to understand the molecular mechanisms of stem cell regulation [71]. The Hippo signalling pathway in *Drosophila,* as it does in other organisms, regulates cell proliferation as well as cell survival in different types of epithelia [72], including the intestine [72], [73]. At least some of the Hippo signalling functions in the *Drosophila* intestinal precursors are mediated by the chromatin-remodelling factor Brahma [73]. The absence of Brahma prevents the cell division and differentiation of intestinal precursors; whilst opposite phenotypes appear in overexpressing conditions. Brahma contains caspase-cleavage sites [73] and uncleavable mutant versions of this protein can sustain continuous cell proliferation [73]. Although the cleavage of Brahma in this system depends on the conventional apoptotic pathway, the caspase activation could be instrumental for limiting the regenerative process (proliferation and differentiation) triggered upon tissue damage [73]. Interestingly the intersection between caspases and the Hippo signalling pathway is likely to occur at different levels. In this sense, there is convincing evidence indicating that Hippo activation normally prevents the upregulation of natural inhibitors of apoptosis [74], while caspases can mediate the enzymatic processing of several Hippo pathway members (e.g. Mst-1) [75]. Importantly the anti-proliferative role of caspases is also observed in mammalian systems [76], [77]. Caspase-1 mutant mice show increased cell proliferation upon chronic injury that leads to higher frequency of tumour formation [76] (Fig. 3A). Intriguingly, this effect is independent from the pro-inflammatory responses normally mediated by caspase-1 [76]. Furthermore biopsies from patients with adenoma or colon carcinoma indicate that caspase-9 deregulation is a common feature that alters the rate of cell division and differentiation of intestinal precursors, and ultimately correlates with bad prognosis [77] (Fig. 3A).

Strikingly, caspase activation can also act as a pro-survival factor under cellular stress and organ damaging conditions. Katarzyna Oficjalska and co-authors have recently observed that caspase-11 mutant mice show an enhanced susceptibility to dextran sodium sulphate treatment, which results in exacerbated cell death and diminished intestinal cell proliferation [78]. The pro-survival function of caspase-11 in this context was attributed to the non-canonical activation of the inflammasome and the altered production of the pro-inflammatory cytokines IL-1β & IL-18 [78]. Furthermore this caspase-protective function does not appear limited to the inflammatory caspases. Caspase-3 knockout mice paradoxically exhibit increased cell death, organ damage and severe colitis in response to various chemical and environmental stresses. This was molecularly associated with the lack of caspase-mediated RasGAP cleavage and the subsequent impaired activation of the pro-survival kinase Akt [79] (Fig. 3A).

### 4.4 About the intimate relation of bone, muscle and skin precursors with caspases

Bone morphogenetic proteins (BMPs) are fundamental for driving the differentiation of osteoblasts (bone cell precursors) [80]. Thus the exposure of the osteoblastic cell line MC3T3-E1 to BMPs induces cell cycle arrest in the G0/G1 transition [80], [81] as well as activation of differentiation markers (alkaline phosphatase (ALP), parathyroid hormone (PTH)-dependent) [80] (Fig. 3A). These effects correlate with the upregulation of several caspase members (caspase −8, −2, −3) without noticeable signs of cell death [80]. Paradoxically, caspase inhibitors can partially alleviate the BMP-induced arrest in the cell cycle [80], but caspase-3 mutant mice show a strong reduction in proliferation and differentiation that, in turn, correlates with the presence of senesce factors (p53, p21) [81]. In caspase-3 knockout mice primary osteoclasts are reduced in number and the NF-κB ligand (RANKL) cannot trigger cell differentiation [82]. Similar results were observed using RAW264.7 cells (mouse leukemic monocyte-macrophages) in the presence of caspase inhibitors [82]. Molecularly it has been described that the withdrawal of caspase activity prevents the translocation of the transcription factor NF-κB into the nucleus, which normally governs the differentiation process of osteoclasts [82]. Recent experiments performed under hypoxic conditions indicates that silencing of caspase-3 in bone marrow mesenchymal stem cells stimulates their proliferation, whilst reducing apoptosis [83]. Although all of this data suggests strong links between caspases and osteogenic networks, the molecular integration of caspase activities and their effects in this cellular scenario remain largely unclear. The most recent research efforts are attempting to elucidate these questions by using high-throughput approaches [84].

Similar to erythroid precursors, a deficiency in caspase-3 activity leads to a dramatic reduction in both myotube/myofibril formation and differentiation of skeletal muscle progenitors (myoblasts) [75] (Fig. 3A and C). The pioneering work of Lynn Megeney laboratory demonstrated for the first time that the non-apoptotic activity of caspase-3 is essential to mediate the proteolytic processing of the Mammalian Sterile Twenty-like kinase (Mst-1) that facilitates the differentiation program of skeletal muscle precursors [75]. More recently the same research group have also shown the cleavage of the Inhibitor of Caspase-Activated Dnase (ICAD) [85] during this process (Fig. 3C). Strikingly ICAD cleavage releases the nuclease Caspase-activated DNase (CAD), which specifically generates double strand breaks in the promoter region of the cell cycle inhibitor p21. These DNA breaks paradoxically trigger the transcriptional activation of p21 [85] (Fig. 3C). The subsequent p21 upregulation can inhibit the activity of CDK-Cyclin complexes essential for progressing through the cell cycle, thus facilitating the completion of the differentiation program [85]. At early stages of the myogenic differentiation program, caspase-3 also cleaves Pax7 (the master regulator of self-renewal in muscle cells), thus initiating the process of cell commitment [86]. Caspase-9 activation precedes the processing of caspase-3 and subsequent exit into the myoblastic differentiation program of C2C12 cells. Furthermore the mitochondrial pathway, which normally triggers apoptosis, seems to be upstream of the caspase-9 activation during skeletal muscle differentiation [87] (Fig. 3C). The differentiation-promoting role of caspases is not restricted to skeletal muscle progenitors and is also observed in cardiomyocyte progenitors [88]. In this context, caspase-3 and caspase-8 activation in response to Wnt-11 facilitates the degradation of β-catenin [88] (Fig. 3C). This ultimately prevents the transcriptional responses triggered by the conventional Wnt signalling pathway and initiates the cardiomyocyte differentiation [88]. Importantly, caspase function is also fundamental for stimulating the differentiation of ESCs into cardiac progenitors whilst concurrently stimulating their proliferation in cell culture conditions (see section 3) [37]. Beyond the cited studies, recent high-throughput transcriptomic/proteomic analyses have begun to decipher the intricate network of signalling pathways regulated by caspase-3 and caspase-7 during heart maturation in mice [38]. Intriguingly, in this study the pro-differentiation effect of caspases is still observed, but fails to identify β-catenin as a direct target of caspase-3 activity [37]. In comparison to previous findings [87], the authors explained this unexpected result through the potential action of a secondary protease that could be responsible for the processing of β-catenin [37]. An alternative to this explanation would be the direct processing of β-catenin mediated by caspase-8 in heart precursors [87], which has been previously described in vitro [89]. However the experimental set up used in the proteomic experiments could not cover this possibility, since it did not include caspase-8. Although these results strongly support the involvement of caspases in the regulation of stem cell physiology in this cellular scenario, further investigations are needed in order to illuminate the molecular details of their implication.

Keratinocytes are the predominant cellular derivatives present in the epidermis. Caspase-3 activation plays a key role in rerouting interfollicular embryonic keratinocytes (cellular precursors of the skin) from proliferation to differentiation [90]. Similarly, caspase-7 deficiency increases mast cell proliferation in hair follicles during early stages of mouse development [91] (Fig. 3A). In addition to normal developmental scenarios, recent investigations have highlighted a crucial contribution of caspase-3 to the progression of oral squamous carcinoma. Caspase-3 upregulation in these cancer cells sustains high rates of cell proliferation, promoting therefore tumorigenesis [92].

## 5 Remote effects of caspase activation in stem cells

Stem cells usually maintain low rates of self-renewal and cellular differentiation. This expands the longevity of their regenerative ability, whilst satisfying the demand for repair after cell loss or tissue wounding. The release of cellular contents and pro-inflammatory signals from apoptotic and damaged cells is one of the major stimulatory mechanisms of stem cell activation. Since these effects are mainly associated with the apoptotic process, they will not be commented in detail in this review [93], [94], but there are several aspects still worth highlighting. The emanating signals from apoptotic cells are crucial for ensuring wound healing and tissue regeneration [93], [94], [95]. This signalling often has a proliferation-promoting effect on both normal differentiated cells and resident stem cells. The nature of these signals frequently involves caspase activation, but does not necessarily requires the completion of the apoptotic program [93], [94], [95], [96]. Caspase activation in such situations conventionally facilitates the release of mitotic signals that can act remotely in neighbouring cells. In some cases the release of signalling molecules require the caspase-mediated proteolytic processing in order to be secreted extracellularly (e.g IL-1 [97]), while in other situations caspase activation only indirectly drives the production of soluble factors (e.g Wnt ligands [98]) or exovesicles [95]. Although these caspase-dependent mechanisms of signalling at a distance are essential for important cellular processes (e.g. apoptosis-induced proliferation, compensatory proliferation and inflammation), they are not yet fully understood. Notably, it has been shown that deregulation of caspase activity in such situations can instigate multiple diseases (cancer, chronic inflammatory disease etc) [93], [94], [96].

## 6 Hypothetical pathological consequences of caspase deregulation in stem cells

As described in previous sections, caspases are involved in the regulation of stem cell properties in a wide variety of adult/embryonic tissues, and therefore, they are prevalent in many cellular processes during development and adulthood. Taking this into account, one can easily envisage that caspase deregulation could be at the origin of many human disorders, particularly in cancer. Dysregulation of caspase-mediated stem cell functions can instigate tumour formation/progression, by generating subsets of proliferative cells that cannot follow their normal route of differentiation (see details above). The malignancy in these cells is aggravated by their inability to undergo cellular death through apoptosis [33], [93], [94]. Importantly, the appearance of such cellular populations in leukaemia and within pre-established solid tumours can fuel tumour progression, forming a highly resistant group of cancer stem cells [33]. Substantial efforts are currently being devoted towards identifying and selectively destroying such cell populations within tumours, as they are considered major risk factors for tumour relapse and bad prognosis [33]. Furthermore, caspase deregulation can facilitate the metastatic behaviour of different cell types in a cellular context dependent manner [21], [22], [23]. Beyond the cell-autonomous effects on stem cells, caspase malfunction could also instigate tumour progression indirectly via mitogenic signals able to act at a distance [93], [94], [95] and/or by altering the inflammatory response [19], [96], [99].

The silencing or aberrant activation of caspases during development usually has fatal consequences, as demonstrated by the early lethality observed in mutant animals for many of the caspase family members [2], [8]. This lethality was initially attributed to defects in apoptosis, but compelling evidence suggests that it is also related with non-apoptotic processes [2]. As reviewed in previous sections, the association between caspases and stem cells could account for severe heart malformations, immunological disorders, neurological defects and epithelial disorders. Paradoxically, the association of specific mutations in caspase genes with human diseases is rare, but caspase malfunction is a common feature of many diseases [8]. This paradox can be explained by the fact that caspase deregulation in disease scenarios is often a secondary consequence of metabolic and signalling defects [8].

## 7 Therapeutic potential of caspase modulation in stem cells

The enzymatic nature of caspases and its connection with the apoptosis programme has incentivised pharmaceutical companies in recent years to identify compounds with caspase-modulating activity. A large proportion of the therapeutic molecules currently available attempt to prevent the inhibitory mechanisms of caspase activation in order to stimulate the cell death of undesired cells [100], [101], [102], [103]. In other cases such molecules are competitive inhibitors of caspase substrates, which prevent apoptotic-dependent tissue damage [104]. Caspase inhibitors have also been used to regulate the inflammatory response [105] and treat specific diseases [106], [107]. However the inclusion of these molecules into the clinic has proven challenging [108], [109], [110]. Compounding the already fine balance between cell survival and cell death, the lack of specificity of caspase-modulating molecules has compromised its clinical application. In addition, the recent implication of caspases in non-cell death related processes has increased the risk of unexpected side effects (i.e. caspase activation could favour the cell death in specific types of tumours, but could also stimulate dangerous metastatic behaviour in others [108]). However new therapeutic strategies using these molecules could emerge, once their involvement in the regulation of stem cell properties is clearly elucidated. In this sense, caspases could be powerful tools in regenerative medicine and tissue repair, since it has been shown that they can facilitate the survival and differentiation of stem cells into specific cell derivatives [37]. Caspases can also aid the reprogramming of differentiated cells into iPSCs [40] and developed engineered tissues [111]. In addition, newly developed sensors suitable for detecting caspase activation in complex biological systems are appearing, and consequently, new experimental opportunities to anticipate the efficacy and toxicity of compounds with caspase-modulating activity [112], [113], [114], [115], [116], [117].

## 8 Concluding remarks

The latest findings pertaining to the evolutionary conserved caspase family indicate their instrumental role in regulating a large cohort of cellular processes other than cell death, including the fine-tuning of stem cell properties. However the molecular mechanisms maintaining the caspase activity at sub-lethal thresholds remain largely unknown. Equally undetermined is the likely vast repertoire of target proteins and substrates modulated by caspases in non-lethal scenarios. Resolution of these questions is fundamental to our understanding of caspase regulation, but also the biological processes in which they participate. Furthermore, banking on this knowledge, novel therapeutic strategies against multiple diseases could emerge. In this regard, specific caspase modulation in stem cells could contribute decisively to the progress of regenerative medicine and tissue repair.

## Contributions

L.A.B-L wrote the original text and contributed to the bibliography search and figure design. L.A and D.C.X contributed to the bibliography search, critical reading of the manuscript, and figure design. A.G participated in the bibliography search and critical reading of the manuscript.
